# Peer review: Heart and soul of scientific publication

**DOI:** 10.4103/0019-5413.45317

**Published:** 2009

**Authors:** Anil K Jain

**Affiliations:** *Editor IJO, Professor, University College of Medical Sciences and Guru Teg Bahadur Hospital, New Delhi - 110 095, India*

The objective of “peer review” is to assist the editors to take a decision about the publication of a manuscript and increase the quality of scientific communications.^1^ It provides the third persons' view about the manuscript to the authors to enhance the quality of article to be published. While writing a manuscript, the vision of author may become tubular, and it is difficult to find faults that a third person can make. To what extent does the peer review and editorial process improve the quality of scientific report is debatable, but it definitely improves the presentation of research. Peer review process provides feed back to the authors and editors of journal and aims to ensure that reader receives valid and scientific information from a publication that is worth the time. It may save an author from the later embarrassment for an untidy publication.

The reviewing of a manuscript is an art for which no formal training is available. It is not taught during postgraduate medical education. However, it can be nurtured and improved with the time and practice.^2^ Attentive peer review is a gift for authors, editors, journal as well for the subject. As an editor, we and all other editors are always looking out for good reviewers who would help us in quality control and process efficiency.^2^

Once a manuscript reaches the hand of an editor, it is triaged at first instance by most of the editors.^1^ The manuscripts that are not following the instructions to authors or do not have conclusions derived from methodologically sound study or do not have a clear research question or new message are sent back to the authors. The rest of the manuscripts are taken up for peer review or “referring.”

## REVIEW PROCESS FOR INDIAN JOURNAL OF ORTHOPAEDICS

Every journal (and in fact, every editor) has its own protocol for performing the peer-review process. We at the Indian Journal of Orthopaedics (IJO), usually allot 3–5 reviewers for a manuscript. Every journal carries a list of subject specific dedicated reviewers. The researchers can volunteer themselves to become a reviewer. The reviewers are chosen from already available list of subject experts or after a “PubMed” search on the subject. They are enrolled and invited electronically. The potential reviewer thus invited has an option to decline the review of the manuscript. If a particular manuscript has a lot of statistical calculations, then a statistician is also involved in the review. The reviewers are generally given three weeks to review the manuscript. Few days before the expiry of three weeks, they receive a reminder email to submit the review. If they still do not submit the review, then another series of reviewers are allotted. Generally, based on the opinions of at least two reviewers, the editor takes a decision. If both the reviews are contradictory to each other in their opinion, then a third reviewer is invited.

## WHAT REVIEWER SHOULD DO ON BEING INVITED?

On receiving an invitation to review a manuscript, the reviewer has to take a decision whether to review it or not. The invited reviewer should not be tempted to accept the invitation. If the reviewer is able to review the manuscript in the specified time frame, does not have any conflict of interest, and if he considers himself to be right person of doing the review, he should accept to review the manuscript. When the reviewer has a definite conflict of interest, he should deny reviewing or should discuss with the editor.

## HOW TO DO THE REVIEW?

Once a manuscript has come for review, the reviewers should read it at the first instance from beginning to end with an emphasis on understanding the research question effectively; another reading after a few days makes the thought process clear. A search of literature on the subject helps in crystallizing the thought process. Most of the website has a “PubMed” link to provide a relevant search. On an average, the review of a manuscript requires 1–3 hours and review needs 500–1000 words.^1^ The manuscript should be evaluated on the following technical and ethical issues.

- Scientific quality of work in terms of proper methodology to conduct the study and whether conclusions are drawn on the basis of results obtained; the variables chosen are proper to the research question or not; the study is prospective or retrospective; if randomized, then how randomization is done; the inclusion or exclusion criteria are explicitly clear or not; how the results are evaluated and whether statistical calculation has been done or not?
- Presentation of the manuscript – whether the manuscript is written in clear, precise language and does the title reflect the content; whether the abstract indicate the purpose of work, what was done, what was found and its significance; does the figures show the intended message and are required to stress the point; and whether the tables and figures need to be condensed/omitted; the relevant references chosen are written in the style of journal and are well cited or not. The discussion section is organized to highlight the strength and limitation of the research. The conclusions drawn should be based on observation and an answer to the research question.
- Ethical concern – if it is a study on human being, whether clearance from institutional review board has been taken or not. When dealing with experimental animals, one must comment whether proper care without any violation about the guidelines to manage the laboratory animal is taken or not.

## SUBMITTING COMMENTS TO THE JOURNAL OFFICE

The comments should provide a rating to manuscript related to the work done on the similar subject. Comments to author should include specific comments on the desired presentation of data, results and discussion. On this column, no recommendation is to be made about acceptability or rejection for publication.

Confidential comments regarding the novelty, significance, strength and weakness of the manuscript are sent to editors. It is to be stated whether the manuscript is suitable for publication and should not be sent to the author.

## RESPONSIBILITY OF THE REVIEWERS

The reviewers are expected to provide a definite reason or appropriate citation and not simply make such remark as “accept it” or “reject it” or ask a leading question doubting the validity of the data.^2^ He should not comment on the integrity of the author. The reviewer should comment on only those aspects of manuscript which he is most familiar with and refrain from commenting outside his field. If any such aspect exists, then it should be intimated clearly.

The reviewer should provide an honest and critical assessment of the manuscript and comments on strength and weakness of the manuscript and may suggest what future work on the subject would improve the quality of manuscript.^2^ He should maintain the confidentially about the existence and the substance of the manuscript. If a reviewer has used the expertise/help of any other colleague, then it should be intimated to the editor. The reviewer should not misuse the data or the language written by reviewers should not be harsh and sarcastic, and the report should be written in a collegial constructive manner. It is most important that reviewer should treat the manuscript in same manner as he wants his manuscript to be treated. If reviewers finds an evidence of duplicate/salami publication or of plagiarism, then it should be reported to the editor. Whole review process depends on the integrity and faith or author, reviewer and editor.

## HOW THE REVIEWERS ARE BENEFITED?

The peer review, traditionally, has been a double blind process where the authors and reviewers do not know each others' identity. With most journals, the reviewer neither obtains remuneration and nor any other direct benefit. They do it for the sense of duty, selflessness and a desire to contribute in an important way to maintain the high standard and veracity in their respective areas of research.^2^ Some journals including IJO publish the list of reviewers every year in their journals.

## SUMMARY

In the end, it is most important to get a proper review for improving and reporting of the research to give crystal clear message. It allows the editor to choose most eligible research to be communicated to the readers, which is worth their time in reading, and indirectly affects the clinical practice, thereby helping in alleviating pain and suffering. It allows author to meet not only the standard of their discipline but also of science in general. The peer review and editorial process helps to identify too much information or too little information or inaccurate information or misplaced information or structural problem, if any, and take remedial measures. Peer review is heart and soul of scientific publishing, and the best reward for a person as a reviewer is his contribution to the quality of published literature. It is an honour and privilege to be selected as a reviewer and to have an opportunity to work cooperatively and constructively as teacher or mentor to the author.^2^ While writing a manuscript, the author just has a similar thought process. The only difference is that our vision gets tubular while writing, and it is difficult to find faults in our own manuscript, while reviewing the faults in the manuscript are efficiently noted.
